# 1-(3-Bromo­phen­yl)thio­urea

**DOI:** 10.1107/S1600536812031601

**Published:** 2012-07-18

**Authors:** Hoong-Kun Fun, Ching Kheng Quah, Prakash S. Nayak, B. Narayana, B. K. Sarojini

**Affiliations:** a X-ray Crystallography Unit, School of Physics, Universiti Sains Malaysia, 11800 USM, Penang, Malaysia; b Department of Studies in Chemistry, Mangalore University, Mangalagangotri 574 199, India; c Department of Chemistry, P. A. College of Engineering, Nadupadavu, Mangalore 574 153, India

## Abstract

In the title compound, C_7_H_7_BrN_2_S, the thio­urea moiety is nearly planar (r.m.s. deviation = 0.004 Å) and it forms a dihedral angle of 66.72 (15)° with the benzene ring. The C—N—C—N2 torsion angle is 15.1 (4)°. In the crystal, mol­ecules are linked *via* N—H⋯S and N—H⋯N hydrogen bonds into sheets lying parallel to (101).

## Related literature

For general background to and related structures of the title compound, see: Fun *et al.* (2012 ▶); Sarojini *et al.* (2007 ▶). For standard bond-length data, see: Allen *et al.* (1987 ▶). For the stability of the temperature controller used for the data collection, see: Cosier & Glazer (1986 ▶).
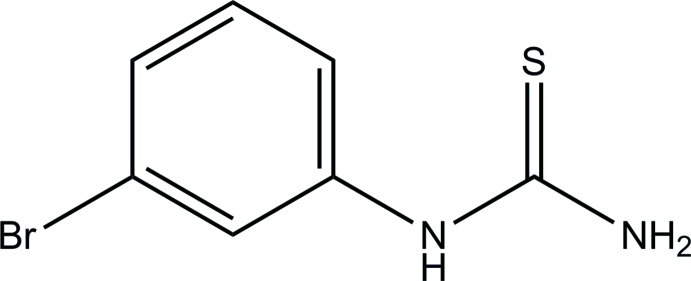


## Experimental

### 

#### Crystal data

C_7_H_7_BrN_2_S
*M* *_r_* = 231.12
Triclinic, 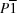

*a* = 5.5308 (8) Å
*b* = 8.5316 (12) Å
*c* = 9.4249 (14) Å
α = 103.500 (3)°
β = 90.878 (3)°
γ = 97.232 (4)°
*V* = 428.54 (11) Å^3^
*Z* = 2
Mo *K*α radiation
μ = 4.97 mm^−1^
*T* = 100 K
0.23 × 0.16 × 0.07 mm

#### Data collection

Bruker SMART APEXII DUO CCD diffractometer
Absorption correction: multi-scan (*SADABS*; Bruker, 2009 ▶) *T*_min_ = 0.396, *T*_max_ = 0.716
5292 measured reflections
1481 independent reflections
1354 reflections with *I* > 2σ(*I*)
*R*_int_ = 0.034

#### Refinement

*R*[*F* ^2^ > 2σ(*F* ^2^)] = 0.024
*wR*(*F* ^2^) = 0.067
*S* = 1.09
1481 reflections
100 parameters
H-atom parameters constrained
Δρ_max_ = 0.44 e Å^−3^
Δρ_min_ = −0.48 e Å^−3^

### 

Data collection: *APEX2* (Bruker, 2009 ▶); cell refinement: *SAINT* (Bruker, 2009 ▶); data reduction: *SAINT*; program(s) used to solve structure: *SHELXTL* (Sheldrick, 2008 ▶); program(s) used to refine structure: *SHELXTL*; molecular graphics: *SHELXTL*; software used to prepare material for publication: *SHELXTL* and *PLATON* (Spek, 2009 ▶).

## Supplementary Material

Crystal structure: contains datablock(s) global, I. DOI: 10.1107/S1600536812031601/hb6892sup1.cif

Structure factors: contains datablock(s) I. DOI: 10.1107/S1600536812031601/hb6892Isup2.hkl

Supplementary material file. DOI: 10.1107/S1600536812031601/hb6892Isup3.cml

Additional supplementary materials:  crystallographic information; 3D view; checkCIF report

## Figures and Tables

**Table 1:** Hydrogen-bond geometry (Å, °)

*D*—H⋯*A*	*D*—H	H⋯*A*	*D*⋯*A*	*D*—H⋯*A*
N1—H1N1⋯S1^i^	1.05	2.28	3.307 (3)	166
N2—H1N2⋯S1^ii^	0.96	2.40	3.349 (3)	168
N2—H2N2⋯Br1^iii^	0.92	2.71	3.468 (2)	141

Symmetry codes: (i) 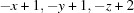; (ii) 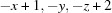; (iii) 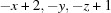.

**Fig. 1.**
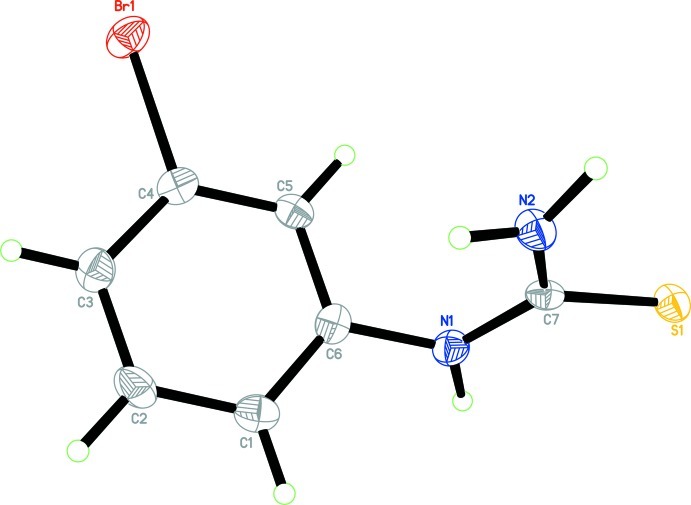
The molecular structure of the title compound showing 50% probability displacement ellipsoids for non-H atoms.

**Fig. 2.**
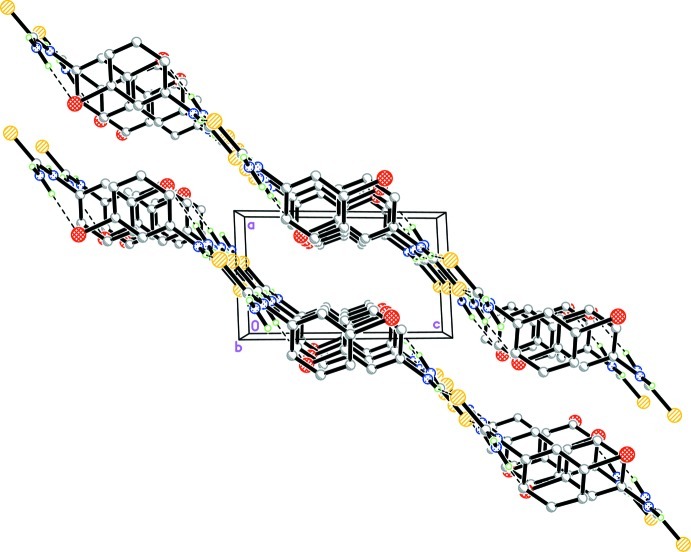
The crystal structure of the title compound, viewed along the b axis. H atoms not involved in hydrogen bonds (dashed lines) have been omitted for clarity.

## supplementary crystallographic information

### Comment

In continuation of our work on synthesis of thiourea derivatives (Fun *et al.*, 2012; Sarojini *et al.*, 2007), the title compound was prepared and its crystal structure is reported here.

In the title molecule (Fig. 1), the thiourea moiety (S1/N1/N2/C7) is nearly planar (r.m.s. deviation = 0.004 Å) and it forms a dihedral angle of 66.72 (15)° with the benzene ring (C1–C6). Bond lengths (Allen *et al.*, 1987) and angles are within normal ranges and are comparable to related structures (Fun *et al.*, 2012; Sarojini *et al.*, 2007).

In the crystal structure, Fig. 2, molecules are linked *via* N1—H1N1···S1, N2—H1N2···S1 and N2—H2N2···Br1 hydrogen bonds (Table 1) into two-dimensional sheets parallel to (101).

### Experimental

3-Bromoaniline (1.39 g, 0.0081 mol) was refluxed with potassium thiocyanate (1.4 g, 0.0142 mol) in 20 ml of water and 1.6 ml of *conc.* HCl for 3 h. The reaction mixture was then cooled to room temperature and stirred overnight. The precipitated product was then filtered, washed with water, dried and recrystallised from ethyl acetate as colourless plates (m.p. = 389–391 K).

### Refinement

N-bound hydrogen atoms were located in a difference Fourier map and refined using a riding model with *U*_iso_(H) = 1.2*U*_eq_(N) [N—H = 0.9156–1.0468 Å]. The remaining H atoms were positioned geometrically and refined using a riding model with C—H = 0.95 Å and *U*_iso_(H) = 1.2*U*_eq_(C).

### Crystal data

**Table:**

C_7_H_7_BrN_2_S	*Z* = 2
*M**_r_* = 231.12	*F*(000) = 228
Triclinic, *P*1	*D*_x_ = 1.791 Mg m^−^^3^
Hall symbol: -P 1	Mo *K*α radiation, λ = 0.71073 Å
*a* = 5.5308 (8) Å	Cell parameters from 3285 reflections
*b* = 8.5316 (12) Å	θ = 2.9–29.6°
*c* = 9.4249 (14) Å	µ = 4.97 mm^−^^1^
α = 103.500 (3)°	*T* = 100 K
β = 90.878 (3)°	Plate, colourless
γ = 97.232 (4)°	0.23 × 0.16 × 0.07 mm
*V* = 428.54 (11) Å^3^	

### Data collection

**Table:**

Bruker SMART APEXII DUO CCD diffractometer	1481 independent reflections
Radiation source: fine-focus sealed tube	1354 reflections with *I* > 2σ(*I*)
Graphite monochromator	*R*_int_ = 0.034
φ and ω scans	θ_max_ = 25.0°, θ_min_ = 2.2°
Absorption correction: multi-scan (*SADABS*; Bruker, 2009)	*h* = −6→6
*T*_min_ = 0.396, *T*_max_ = 0.716	*k* = −10→10
5292 measured reflections	*l* = −11→11

### Refinement

**Table:**

Refinement on *F*^2^	Primary atom site location: structure-invariant direct methods
Least-squares matrix: full	Secondary atom site location: difference Fourier map
*R*[*F*^2^ > 2σ(*F*^2^)] = 0.024	Hydrogen site location: inferred from neighbouring sites
*wR*(*F*^2^) = 0.067	H-atom parameters constrained
*S* = 1.09	*w* = 1/[σ^2^(*F*_o_^2^) + (0.0373*P*)^2^ + 0.1495*P*] where *P* = (*F*_o_^2^ + 2*F*_c_^2^)/3
1481 reflections	(Δ/σ)_max_ = 0.001
100 parameters	Δρ_max_ = 0.44 e Å^−^^3^
0 restraints	Δρ_min_ = −0.48 e Å^−^^3^

### Special details

**Table:**

Experimental. The crystal was placed in the cold stream of an Oxford Cryosystems Cobra open-flow nitrogen cryostat (Cosier & Glazer, 1986) operating at 100.0 (1) K.
Geometry. All esds (except the esd in the dihedral angle between two l.s. planes) are estimated using the full covariance matrix. The cell esds are taken into account individually in the estimation of esds in distances, angles and torsion angles; correlations between esds in cell parameters are only used when they are defined by crystal symmetry. An approximate (isotropic) treatment of cell esds is used for estimating esds involving l.s. planes.
Refinement. Refinement of F^2^ against ALL reflections. The weighted R-factor wR and goodness of fit S are based on F^2^, conventional R-factors R are based on F, with F set to zero for negative F^2^. The threshold expression of F^2^ > 2sigma(F^2^) is used only for calculating R-factors(gt) etc. and is not relevant to the choice of reflections for refinement. R-factors based on F^2^ are statistically about twice as large as those based on F, and R-factors based on ALL data will be even larger.

### Fractional atomic coordinates and isotropic or equivalent isotropic displacement parameters (Å^2^)

**Table:**

	*x*	*y*	*z*	*U*_iso_*/*U*_eq_	
Br1	0.80456 (5)	0.08100 (4)	0.30695 (3)	0.02513 (13)	
S1	0.39118 (12)	0.25378 (9)	1.02969 (8)	0.01925 (18)	
N1	0.7109 (4)	0.3643 (3)	0.8568 (3)	0.0188 (5)	
H1N1	0.6478	0.4767	0.8945	0.023*	
N2	0.7347 (4)	0.1050 (3)	0.8821 (3)	0.0201 (5)	
H1N2	0.6763	0.0004	0.8990	0.024*	
H2N2	0.8746	0.1112	0.8318	0.024*	
C1	1.0878 (5)	0.4459 (4)	0.7452 (3)	0.0208 (6)	
H1A	1.1426	0.5220	0.8340	0.025*	
C2	1.2308 (5)	0.4300 (4)	0.6236 (3)	0.0241 (7)	
H2A	1.3836	0.4966	0.6297	0.029*	
C3	1.1532 (5)	0.3180 (4)	0.4929 (3)	0.0220 (6)	
H3A	1.2529	0.3057	0.4108	0.026*	
C4	0.9281 (5)	0.2254 (4)	0.4859 (3)	0.0185 (6)	
C5	0.7812 (5)	0.2390 (3)	0.6044 (3)	0.0174 (6)	
H5A	0.6265	0.1744	0.5972	0.021*	
C6	0.8650 (5)	0.3495 (4)	0.7348 (3)	0.0183 (6)	
C7	0.6287 (5)	0.2392 (3)	0.9158 (3)	0.0164 (6)	

### Atomic displacement parameters (Å^2^)

**Table:**

	*U*^11^	*U*^22^	*U*^33^	*U*^12^	*U*^13^	*U*^23^
Br1	0.02112 (19)	0.0317 (2)	0.01968 (19)	0.00583 (13)	−0.00184 (11)	−0.00064 (13)
S1	0.0192 (4)	0.0159 (4)	0.0237 (4)	0.0044 (3)	0.0047 (3)	0.0055 (3)
N1	0.0211 (12)	0.0164 (13)	0.0189 (13)	0.0044 (10)	0.0024 (10)	0.0029 (10)
N2	0.0210 (12)	0.0149 (13)	0.0256 (13)	0.0053 (10)	0.0052 (10)	0.0057 (10)
C1	0.0207 (15)	0.0199 (16)	0.0220 (15)	0.0030 (12)	−0.0053 (12)	0.0055 (12)
C2	0.0155 (15)	0.0282 (18)	0.0283 (17)	−0.0024 (13)	−0.0029 (13)	0.0092 (14)
C3	0.0163 (15)	0.0281 (17)	0.0224 (15)	0.0058 (13)	0.0008 (12)	0.0061 (13)
C4	0.0181 (14)	0.0192 (16)	0.0185 (14)	0.0064 (12)	−0.0030 (11)	0.0036 (12)
C5	0.0161 (14)	0.0140 (15)	0.0230 (15)	0.0033 (11)	−0.0007 (11)	0.0052 (11)
C6	0.0194 (14)	0.0198 (15)	0.0179 (15)	0.0065 (12)	0.0011 (11)	0.0069 (12)
C7	0.0169 (14)	0.0135 (14)	0.0176 (14)	0.0007 (11)	−0.0044 (11)	0.0025 (11)

### Geometric parameters (Å, º)

**Table:**

Br1—C4	1.900 (3)	C1—C2	1.393 (4)
S1—C7	1.707 (3)	C1—H1A	0.9500
N1—C7	1.348 (4)	C2—C3	1.395 (4)
N1—C6	1.433 (4)	C2—H2A	0.9500
N1—H1N1	1.0468	C3—C4	1.380 (4)
N2—C7	1.327 (4)	C3—H3A	0.9500
N2—H1N2	0.9608	C4—C5	1.383 (4)
N2—H2N2	0.9156	C5—C6	1.395 (4)
C1—C6	1.381 (4)	C5—H5A	0.9500
			
C7—N1—C6	123.6 (2)	C2—C3—H3A	120.9
C7—N1—H1N1	119.2	C3—C4—C5	122.0 (3)
C6—N1—H1N1	116.9	C3—C4—Br1	120.0 (2)
C7—N2—H1N2	127.4	C5—C4—Br1	117.9 (2)
C7—N2—H2N2	116.3	C4—C5—C6	118.6 (3)
H1N2—N2—H2N2	116.2	C4—C5—H5A	120.7
C6—C1—C2	119.0 (3)	C6—C5—H5A	120.7
C6—C1—H1A	120.5	C1—C6—C5	121.0 (3)
C2—C1—H1A	120.5	C1—C6—N1	120.6 (3)
C1—C2—C3	121.1 (3)	C5—C6—N1	118.3 (3)
C1—C2—H2A	119.5	N2—C7—N1	118.5 (3)
C3—C2—H2A	119.5	N2—C7—S1	121.2 (2)
C4—C3—C2	118.3 (3)	N1—C7—S1	120.3 (2)
C4—C3—H3A	120.9		
			
C6—C1—C2—C3	−0.5 (4)	C2—C1—C6—N1	−178.9 (2)
C1—C2—C3—C4	1.4 (4)	C4—C5—C6—C1	1.3 (4)
C2—C3—C4—C5	−1.0 (4)	C4—C5—C6—N1	179.3 (2)
C2—C3—C4—Br1	176.0 (2)	C7—N1—C6—C1	−123.6 (3)
C3—C4—C5—C6	−0.3 (4)	C7—N1—C6—C5	58.4 (4)
Br1—C4—C5—C6	−177.34 (19)	C6—N1—C7—N2	15.1 (4)
C2—C1—C6—C5	−0.9 (4)	C6—N1—C7—S1	−164.2 (2)

### Hydrogen-bond geometry (Å, º)

**Table:**

*D*—H···*A*	*D*—H	H···*A*	*D*···*A*	*D*—H···*A*
N1—H1*N*1···S1^i^	1.05	2.28	3.307 (3)	166
N2—H1*N*2···S1^ii^	0.96	2.40	3.349 (3)	168
N2—H2*N*2···Br1^iii^	0.92	2.71	3.468 (2)	141

Symmetry codes: (i) −*x*+1, −*y*+1, −*z*+2; (ii) −*x*+1, −*y*, −*z*+2; (iii) −*x*+2, −*y*, −*z*+1.
